# Assessing the Biodegradation of Lignin/PBAT Composites: A Comparative Study of Weight Loss and Mineralization

**DOI:** 10.3390/polym18162010

**Published:** 2026-08-18

**Authors:** Yanyan Dong, Shaochuang Su, Hong Yang, Zixi Han, Dan Huang, Xiaoshuai Han, Mingqiang Zhu, Fangda Zhang

**Affiliations:** 1 Key Laboratory for Prevention and Control of Residual Pollution in Agricultural Film, Ministry of Agriculture and Rural Affairs, Institute of Environment and Sustainable Development in Agriculture, Chinese Academy of Agricultural Sciences, Beijing 100081, China; dongyanyan@caas.cn (Y.D.); hanzixi@caas.cn (Z.H.); 2 National Engineering Research Center of Engineering and Ecological Plastics, Technical Institute of Physics and Chemistry, Chinese Academy of Sciences, Beijing 100190, China; su19862096041@163.com (S.S.); yh1145299902@163.com (H.Y.); 3 College of Materials Science and Technology, Nanjing Forestry University, Nanjing 210037, China; xiaoshuai.han@njfu.edu.cn; 4 Xinjiang Sizhong Biotechnology Co., Ltd., Wujiaqu 831300, China; zmqsx@nwsuaf.edu.cn; 5 Research Institute of Wood Industry, Chinese Academy of Forestry, Beijing 100091, China

**Keywords:** PBAT, lignin, degradation performance, weight loss, mineralization rate

## Abstract

Lignin is a promising bio-based filler for poly(butylene adipate-co-terephthalate) (PBAT), yet its true contribution to biodegradation remains unclear—most studies rely on weight loss alone, neglecting CO_2_ mineralization and lignin’s antibacterial activity. Here, lignin/PBAT composites with 1 wt% (LP-1) and 3 wt% (LP-2) lignin are assessed under composting conditions using both weight loss and mineralization rate. Lignin exhibits a dual role: it promotes hydrolytic weight loss (18.3% and 28.6% for LP-1 and LP-2 at 40 days), but significantly inhibits ultimate mineralization (one-year mineralization: 65.71% for pure PBAT vs. 48.74% and 29.53% for LP-1 and LP-2). A negative initial mineralization rate suggests transient antibacterial activity from released phenolic compounds. Lignin increases crystallization temperature (48.4 → 92.6 °C) and *T*_g_ (−28.5 → 49.1 °C), with 1% loading largely preserving mechanical properties. These findings demonstrate that weight loss alone is insufficient to assess degradation; mineralization must be included. Appropriate lignin loading enables balanced performance and degradation controllability, offering new insights into lifecycle assessment of green composites.

## 1. Introduction

Plastic pollution has emerged as a pressing global environmental challenge. In 2022, more than 175 countries passed a resolution at the resumed fifth session of the United Nations Environment Assembly, mandating action across the entire life cycle of plastics—production, design, and disposal—to curb their leakage into ecosystems. Biodegradable plastics are seen as a key solution to conventional plastic pollution. Among them, poly(butylene adipate-co-terephthalate) (PBAT), a fully biodegradable aliphatic–aromatic copolyester, offers high elongation at break, good flexibility, and excellent processability and can be rapidly degraded by microbial enzymes under soil or composting conditions [1,2,3,4,5]. As a result, PBAT is widely used in disposable film products, such as packaging bags, mulch films, and garbage bags, and is considered an ideal substitute for polyethylene (PE) [6,7,8,9]. Nevertheless, large-scale adoption of PBAT is hindered by two major bottlenecks: its relatively high cost—roughly three to five times that of PE—and its inadequate mechanical and thermomechanical properties (e.g., elastic modulus and heat distortion temperature) for certain applications [10,11].

To address these limitations, researchers have incorporated low-cost fillers—including organic ones such as starch [12,13], soybean meal [14], protein [15], rice flour [16], and lignin [17,18], as well as inorganic ones like clay [19] and talc [20]—into the PBAT matrix. This strategy reduces production costs and improves material properties while maintaining biodegradability. Among these, lignin stands out as a highly promising bio-based filler [21]. As the second most abundant biomass resource in nature (accounting for 15–30 wt% of lignocellulose), lignin offers low cost, low density, good thermal stability, and abundant reactive hydroxyl groups.

The hydroxyl and other reactive groups in lignin provide sites for chemical modification to meet diverse application needs, making lignin an ideal filler for promoting interactions between the polymer matrix and lignin. Consequently, most current research has focused on modified lignin systems, with an emphasis on mechanical and thermal properties, whereas evaluations of degradation performance remain relatively limited [17,22,23,24,25]. For instance, Jie Yu et al. incorporated vinyltrimethoxysilane (VTMS)-grafted lignin (VL) into PBAT and reported that the composite containing 30 wt% VL exhibited increases in tensile strength and Young’s modulus by 200% and 151%, respectively [18]. Notably, during soil degradation, increasing VL content accelerated the reduction in PBAT molecular weight and the decay of mechanical properties, leading to the conclusion that lignin promotes PBAT degradation. However, this only indicates that the introduction of lignin accelerates the initial hydrolysis of PBAT, a property expected for most blends containing hydrophilic fillers compared to neat polyesters. In another study, Srimala Sreekantan et al. incorporated acrylic acid-grafted rice husk lignin into PBAT and found that a lignin-to-PBAT ratio of 30:70 gave the best combination of mechanical properties and biodegradability [26]. The weight-loss-based degradation rate reached 57.36% within six months in soil, significantly higher than the 9.2% observed for pure PBAT.

Even unmodified lignin blended with PBAT also showed accelerated degradation. Hengfei Qin et al. prepared mulch films by hot-pressing PBAT with alkali lignin (AL) [27]. At an AL loading of 20 wt%, the composite film (PBAT/AL20) achieved a tensile stress of 22.41 MPa and an elongation at break of 252.35%, and the mass loss rate after 180 days outdoors reached 84.27%. Importantly, the PBAT/AL20 film exhibited a certain ability to inhibit bacterial growth. This is because the polyphenolic compounds in lignin can damage bacterial cell walls and significantly suppress cell viability [28]; in addition, the three-dimensional structure of lignin inhibits bacterial aggregation on its surface [29].

Taken together, previous studies on the biodegradation performance of lignin/PBAT composites—whether using modified or unmodified lignin—have generally relied on measurements such as weight loss of the residual polymer [26,27,30,31,32,33], changes in mechanical properties [18], or molecular weight reduction [18]. These approaches often neglect the transformation of final products. However, the generation of final products (e.g., CO_2_) may actually be suppressed by the antibacterial activity of lignin. Thus, our understanding of the actual contribution of lignin to the biodegradation performance of PBAT is still incomplete.

Therefore, the present study aims to provide a comprehensive evaluation of the degradation behavior of lignin/PBAT composites by simultaneously employing two complementary indicators: weight loss (reflecting physical disintegration) and mineralization rate (reflecting ultimate carbon conversion to CO_2_) under identical composting conditions. Unmodified lignin was blended with PBAT via melt compounding followed by film blowing, and the effects of low lignin loadings (1 wt% and 3 wt%) on mechanical properties, thermal behavior, and biodegradability were systematically investigated. Our findings provide a new perspective on the true role of natural fillers in biodegradable plastics and offer essential fundamental data for the development of low-cost, simple-process, and environmentally friendly lignin/PBAT composites.

## 2. Materials and Methods

### 2.1. Materials

PBAT (a random copolymer of adipic acid, 1,4-butanediol, and terephthalic acid) was purchased from JinhuiZhaolong High Technology Co., Ltd. (Xiaoyi, China) with the trade name of Ecoworld^®^. It possessed a density of 1.26 g/cm^3^, a melt index of 3–5 g/10 min (190 °C/2.16 kg), and an average melting temperature of 115 °C. Lignin was provided by the Beijing Key Laboratory of Lignocellulosic Chemistry, Beijing Forestry University (Beijing, China), with its commercial raw material sourced from Shandong Longlive Bio-Technology Group Co., Ltd. (Dezhou, China). The lignin had a purity of 95%, a weight-average molecular weight (*M*_w_) of 2029 Da, and a dispersity (Ð) of 2.22 [34]. Elemental analysis showed that the contents of carbon, hydrogen, and nitrogen were 57.76%, 4.41%, and 0.33% by mass, respectively [35].

### 2.2. Fabrication of Lignin/PBAT Composite Films

Lignin/PBAT composite films were prepared via melt compounding followed by cast film extrusion. The compositions of all samples are listed in Table 1. PBAT was dried in a vacuum oven at 80 °C for 24 h before use. Then, PBAT was mixed with different contents of lignin (1 and 3 wt%) in a plastic mixer. The mixtures were melt-compounded using a twin-screw extruder (LSF-26, Labtech Engineering Co. Ltd., Samutprakarn, Thailand) at temperatures ranging from 145 °C−180 °C. The screw speed was maintained at 150 rpm, and the feed rate was 3 kg/h, giving a residence time of approximately 2 min. The extruded strands were air-cooled and pelletized. The obtained pellets were subsequently extruded into films using a cast film extruder (LF-400-COEX, Labtech Engineering, Thailand) at 160 °C. The resulting films were designated as LP-0, LP-1, and LP-2, corresponding to lignin contents of 0, 1, and 3 wt%, respectively. All films had a thickness of 22–24 μm, with no significant difference among the three groups.

### 2.3. Composting Degradation

**Figure 1:**
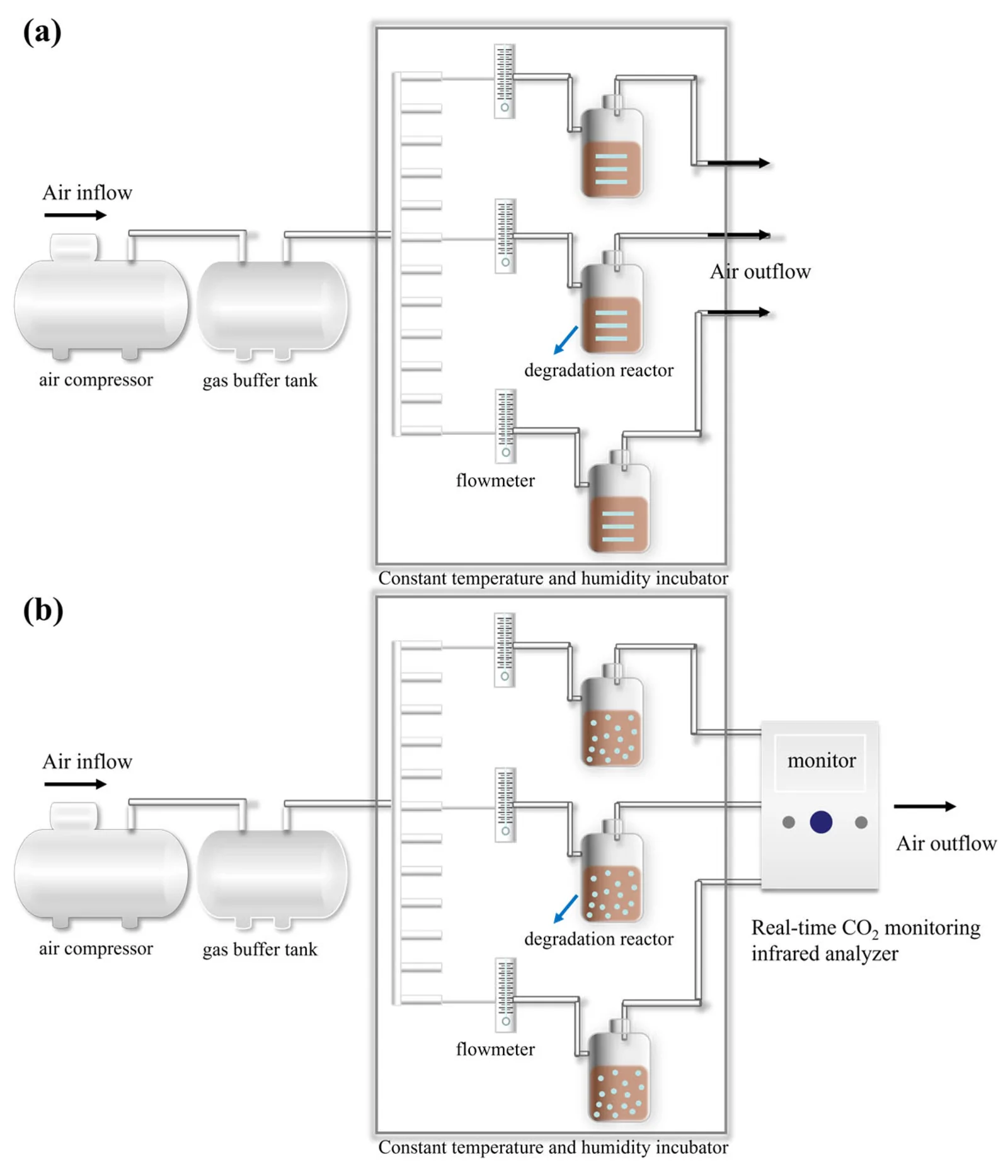
Schematic diagram of the composting degradation experiments: (**a**) film burial in compost; (**b**) mineralization rate testing system.

The composting degradation experiment was conducted following ISO 14855-1:2005 [36]. Two parallel evaluation methods were employed: (i) weight loss of film samples after burial in compost (Figure 1a) and (ii) mineralization rate based on CO_2_ evolution from powder samples (Figure 1b).

The same activated compost inoculum was used for both evaluation methods. The inoculum for composting was prepared according to ISO 14855-1:2005 [36]. Specifically, a culture medium composed of mineral solution, nutrients, urea, corn starch, cellulose, and compost extract was mixed with vermiculite at a volume-to-mass ratio of 3:1 and the mixture was incubated at 48 °C for 48 h to activate the microbial population.

#### 2.3.1. Weight Loss (Film Burial Test)

The weight loss of film samples during composting was determined gravimetrically. Film specimens were cut into squares of 5 cm × 5 cm. Three replicate specimens were taken for each sampling time. Then these specimens were buried in the composting medium. At predetermined time intervals, the recovered films were cleaned, dried, and weighed. The weight loss ratio was calculated using Equation (1):

(1) $$\mathrm{Weight}\;\mathrm{loss}\;(\%)=\frac{W_{0}-W_{t}}{W_{0}}\times 100\%,$$

where W_0_ denotes the mass before degradation and W_t_ represents the mass after degradation.

#### 2.3.2. Mineralization Rate (CO_2_ Evolution)

For the blank control group, no test sample was added. For the mineralization test, 50 g of sample powder was mixed with the activated compost medium and loaded into the degradation reactor. The continuous saturated airflow was introduced at a fixed flow rate to support microbial activity. The airflow and humidity in each degradation reactor were monitored in real time, and deionized water was regularly added while stirring the mixture. The CO_2_ released by microorganisms using the sample as a carbon source for metabolism was quantified using a CO_2_ analyzer. The mineralization rates of the test samples and references were calculated using Equations (2) and (3):

(2) $$Dt\;(\%)=\frac{{\left({CO}_{2}\right)}_{T}-{\left({CO}_{2}\right)}_{B}}{ThCO_{2}}\times 100\%,$$

(3) $$ThCO_{2}=\frac{M_{TOT}\times C_{TOT}\times 44}{12},$$

where (CO_2_)_T_ denotes the average mass of CO_2_ (g) released from the test sample, (CO_2_)_B_ represents the average mass of CO_2_ (g) released from the blank control group, ThCO_2_ denotes the theoretical CO_2_ emission of the test sample, M_TOT_ represents the total dry weight of the test sample (g), and C_TOT_ denotes the total organic carbon content (%).

### 2.4. Characterization

#### 2.4.1. Mechanical Property Testing

Mechanical properties of the films were tested using a universal testing machine (XLW, Labthink, Jinan, Shandong, China). Tensile properties were determined following ASTM D882-2018. Films were cut into 10 mm wide strips at least 80 mm long. The grip separation and crosshead speed were set at 50 mm and 500 mm/min, respectively. Tear properties were determined following ASTM D1004-2013 at a strain rate of 200 mm/min. Film thickness was measured according to ASTM D6988-2013 using a micrometer (C1200, Mahr Millimar, Göttingen, Germany) with an accuracy of ±0.1 µm. At least five specimens were tested for each sample.

#### 2.4.2. Scanning Electron Microscopy (SEM)

The surface morphologies of lignin/PBAT composite films before and after degradation were examined using a SEM (Hitachi S-4800, Hitachi High Technologies America, Inc., Schaumburg, IL, USA). All samples were coated with gold using an ion sputter coater.

#### 2.4.3. FT-IR Spectroscopy (FTIR)

FT-IR spectroscopy for lignin/PBAT composite films was measured using a Micro-Attenuated Total Reflection Fourier Transform Infrared (Micro-ATR-FTIR) microscope (OPUS 7.5, Bruker, Germany). The FTIR spectrum of each sample was obtained at 4000–400 cm^−1^ with a resolution of 4 cm^−1^.

#### 2.4.4. Differential Scanning Calorimetry (DSC)

All samples were measured using a TA DSC Q200 calorimeter under a N_2_ atmosphere at 10 °C/min. A three-step heating/cooling/heating cycle was used: first heating from room temperature to 200 °C, holding for 3 min to eliminate the thermal history, then cooling to room temperature, and, finally, reheating to 200 °C to obtain the second heating curve.

#### 2.4.5. Thermogravimetric Analysis (TGA)

TG analysis was carried out using a TGA/DSC thermogravimetric analyzer (Mettler Toledo Corporation, Greifensee, Switzerland) within a temperature range from room temperature to 800 °C at a heating rate of 10 °C/min under a nitrogen atmosphere.

## 3. Results and Discussion

### 3.1. Mechanical Properties and Compatibility

Lignin possesses a three-dimensional network structure and abundant polar functional groups (e.g., phenolic and aliphatic hydroxyl groups). When directly blended with non-polar PBAT, interfacial incompatibility readily occurs, leading to decreased stress transfer efficiency and reduced mechanical properties. Therefore, in this study, only low lignin loadings (1 wt% and 3 wt%) were employed to minimize the negative effects of incompatibility.

**Figure 2:**
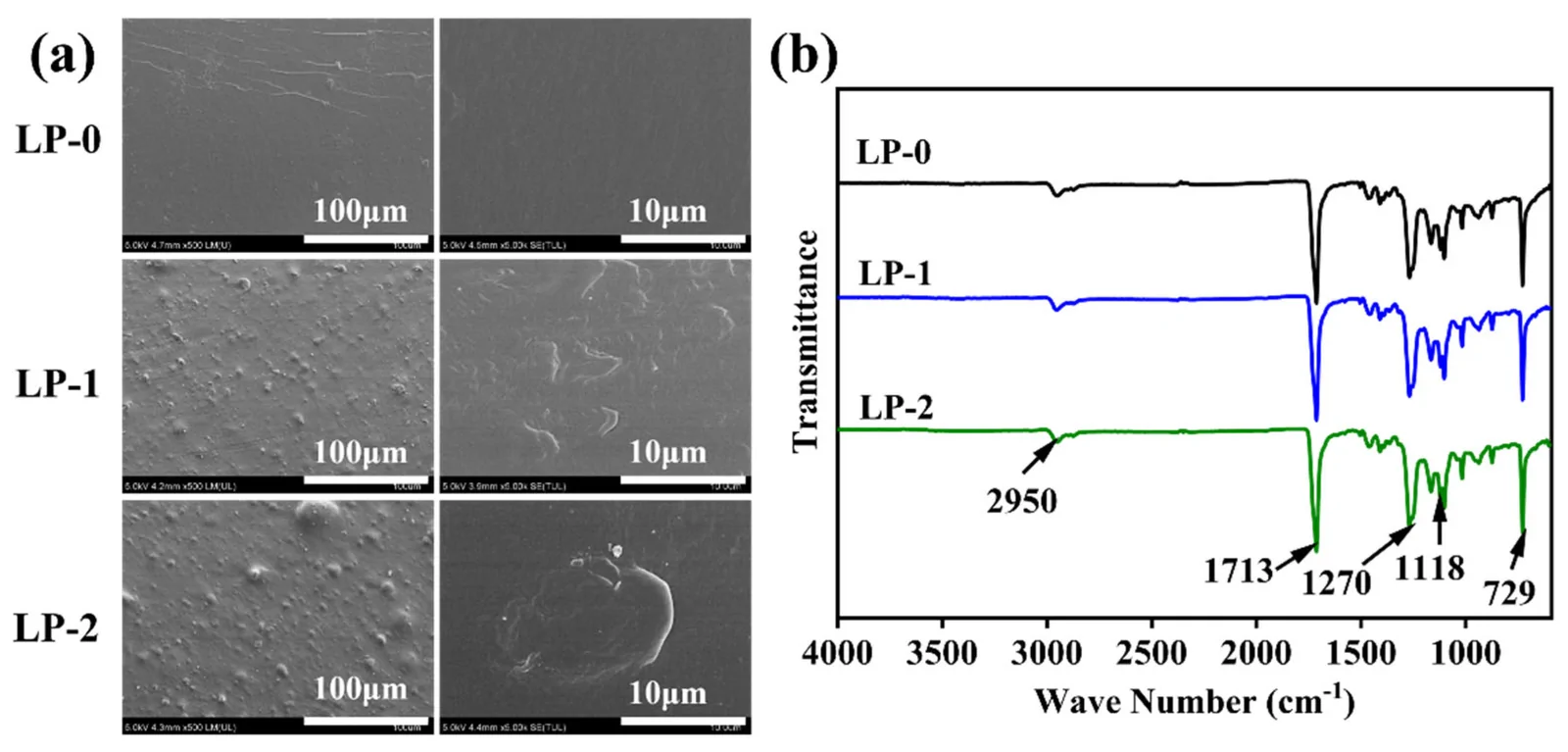
SEM images (**a**) and FTIR spectra (**b**) of samples fabricated with different lignin/PBAT weight ratios.

Table 2 summarizes the mechanical properties of the lignin/PBAT composites. All measured standard deviations fall within acceptable ranges, confirming good repeatability and reliability of the test results. Compared with neat PBAT (LP-0), the composite containing 1 wt% lignin (LP-1) largely maintains the original tensile strength and toughness. Its tensile strengths in the vertical and horizontal directions are 28.4 MPa and 19.8 MPa, respectively, while the corresponding elongations at break are 485.7% and 511.3%. Notably, the tear resistance of LP-1 increases by 0.3 N (vertical) and 0.4 N (horizontal), indicating that the introduction of a small amount of lignin may exert a mild reinforcing effect, which is likely associated with the uniform dispersion of lignin particles within the PBAT matrix. SEM observations (Figure 2a) were used to examine the surface morphology. Compared with the smooth surface of neat PBAT, the surface of LP-1 is slightly rougher: small protrusions are observable at a scale of 100 μm, and faint phase boundaries are visible at a scale of 10 μm. These morphological features, combined with the mechanical data, suggest that the well-dispersed lignin particles at low loading facilitate local stress transfer without significantly compromising mechanical performance.

When the lignin content is further increased to 3 wt% (LP-2), the elongation at break remains essentially unchanged, while the tensile strength decreases slightly (Table 2). Interestingly, the tear resistance in the vertical direction still shows a slight enhancement, but that in the horizontal direction exhibits a more pronounced decrease. This anisotropic behavior may be related to the orientation of lignin particles during cast film extrusion: the interfacial adhesion in the horizontal direction is more susceptible to the aggregation of lignin at higher loadings. SEM images further confirm that as the lignin content increased, the surface protrusions become larger and the phase boundaries become more distinct. Based on these surface morphological differences, it is inferred that the compatibility between lignin and PBAT decreases at higher lignin contents, which, in turn, adversely affects the mechanical properties.

FTIR analysis was performed to examine possible chemical interactions between lignin and PBAT. As shown in Figure 2b, all samples exhibit similar absorption peaks characteristic of PBAT. The peaks near 2930–2950 cm^−1^ correspond to the aliphatic C–H stretching vibration, the absorption peak at approximately 1713 cm^−1^ is assigned to the C=O stretching vibration of PBAT, the peaks at 1270 cm^−1^ and 1010 cm^−1^ are attributed to the C–O–C stretching absorptions, and the peak near 729 cm^−1^ represents the C–H bending vibration of the benzene ring. Additionally, the peaks at 1118 and 1027 cm^−1^ are indicative of the in-plane deformation of aromatic C-H from syringyl (S) and guaiacyl (G) structures, respectively. No significant peak shifts or new bands were observed upon lignin incorporation (1 wt% and 3 wt%), suggesting that the introduction of low lignin loadings did not alter the chemical structure of PBAT. The absence of spectral changes may have been due to the low lignin content, which could have led to detection limitations.

### 3.2. Thermal Properties

**Figure 3:**
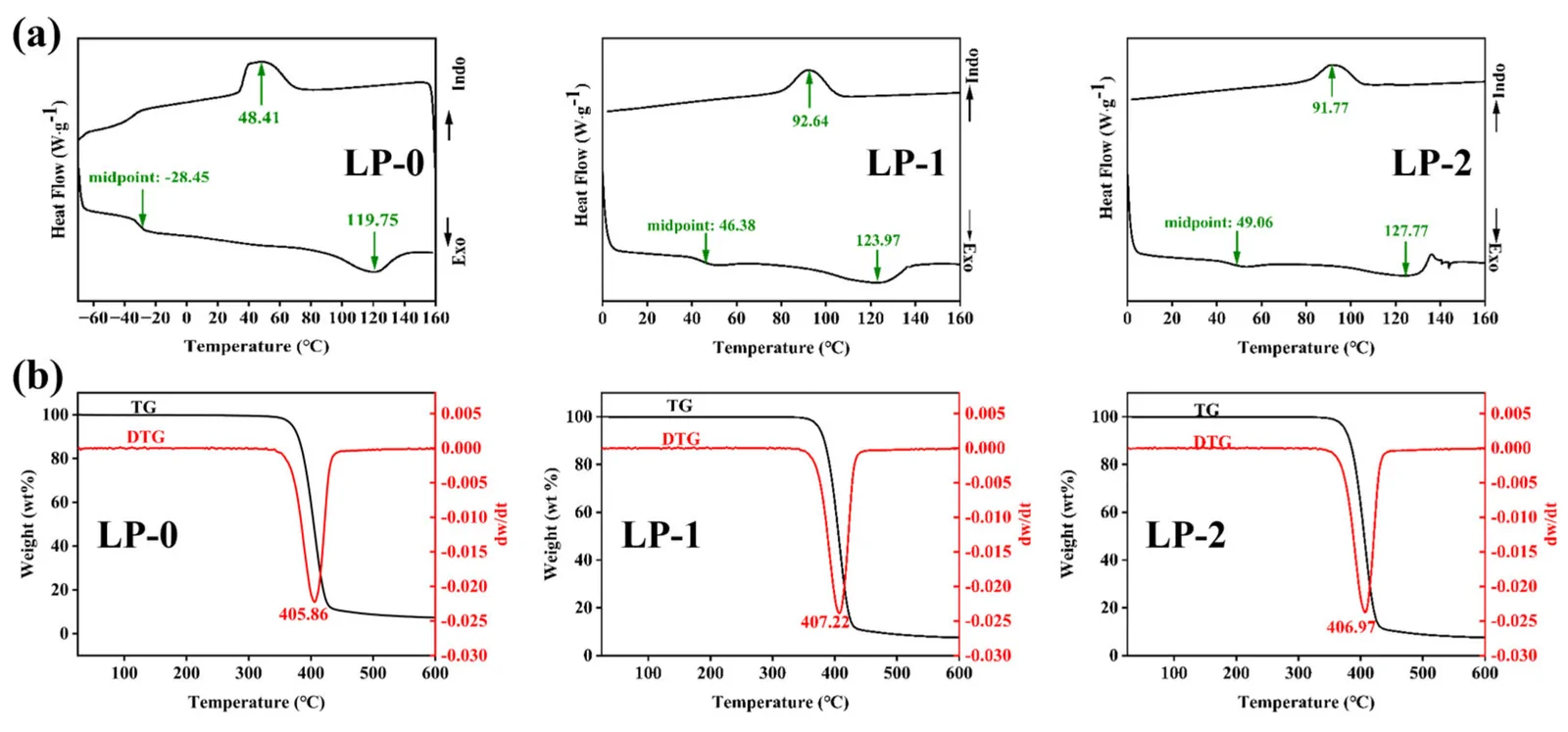
(**a**) DSC cooling and second heating curves and (**b**) TG/DTG curves for samples with different lignin/PBAT weight ratios.

The melting and crystallization behaviors of the lignin/PBAT composites were analyzed by DSC, and the results are shown in Figure 3a and Table 3. Compared with neat PBAT, the crystallization temperatures (*T*_c_) of the composites increase significantly from 48.4 °C to 92.6 °C (LP-1) and 91.8 °C (LP-2) upon lignin incorporation, while the crystallization enthalpy (Δ*H*_c_) decreases from 11.6 J·g^−1^ to 10.9 J·g^−1^ and 10.6 J·g^−1^, respectively. The simultaneous increase in $T_{c}$ and decrease in $\Delta H_{c}$ suggests a more complex crystallization behavior than a simple nucleation effect. We propose that multiple factors are involved: (i) at elevated temperatures, lignin particles may act as heterogeneous nucleation sites, promoting the ordered arrangement of PBAT molecular chains and raising the crystallization onset temperature; (ii) however, as crystallization proceeds, the rigid lignin particles impose spatial confinement and restrict chain segmental mobility, as evidenced by the substantial increase in glass transition temperature ($T_{g}$) from −28.5 °C for pure PBAT to 49.1 °C for LP-2; and (iii) strong interfacial interactions, likely involving hydrogen bonding between lignin hydroxyl groups and PBAT ester groups, may compete with PBAT chain folding during crystal growth, further suppressing the overall crystallinity. This combined effect leads to the formation of fewer but thicker or more perfect crystallites, which require higher temperatures to melt, consistent with the observed shift of the melting temperature ($T_{m}$) from 119.8 °C (LP-0) to 127.8 °C (LP-2), accompanied by a decrease in the melting enthalpy (Δ*H*_m_).

The thermal stability of the composites was further evaluated by TGA, as shown in Figure 3b and Table 3. The results show that the addition of lignin has a slight effect on the thermal decomposition temperatures. For example, the temperature at 5% weight loss (T_d_, 95%) remains nearly unchanged, while the maximum degradation temperature (T_max_) increased from 405.9 °C to 407.2 °C and 407.0 °C for LP1 and LP2, respectively. These findings suggest that the incorporation of lignin retards the thermal degradation of PBAT and enhances its thermal stability. This improvement can be attributed to the inherently high thermal stability and abundant aromatic ring structures of lignin, which facilitate the formation of a char layer at elevated temperatures, serving as a physical barrier that hinders the thermal decomposition of the polymer matrix.

### 3.3. Composting Degradation Performance

**Figure 4:**
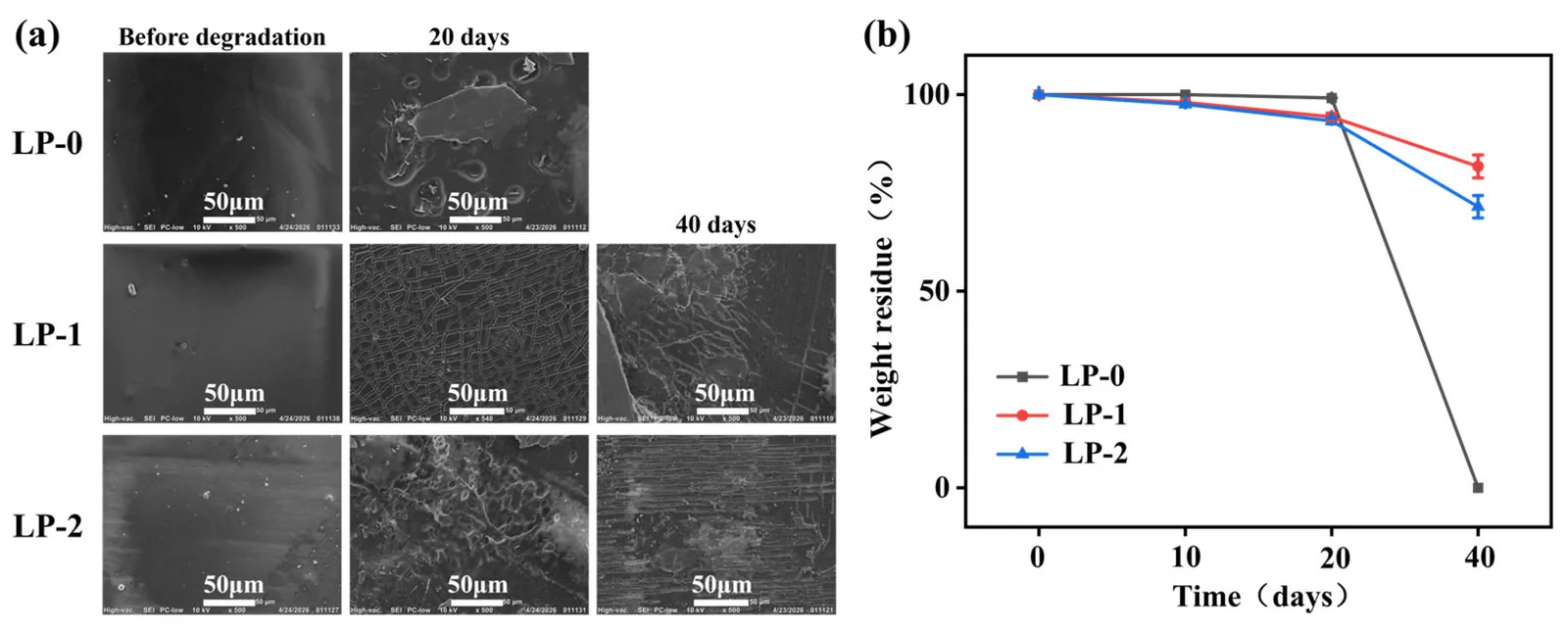
(**a**) SEM images of lignin/PBAT composite films before and after composting degradation; (**b**) weight loss curves of the films during composting.

**Figure 5:**
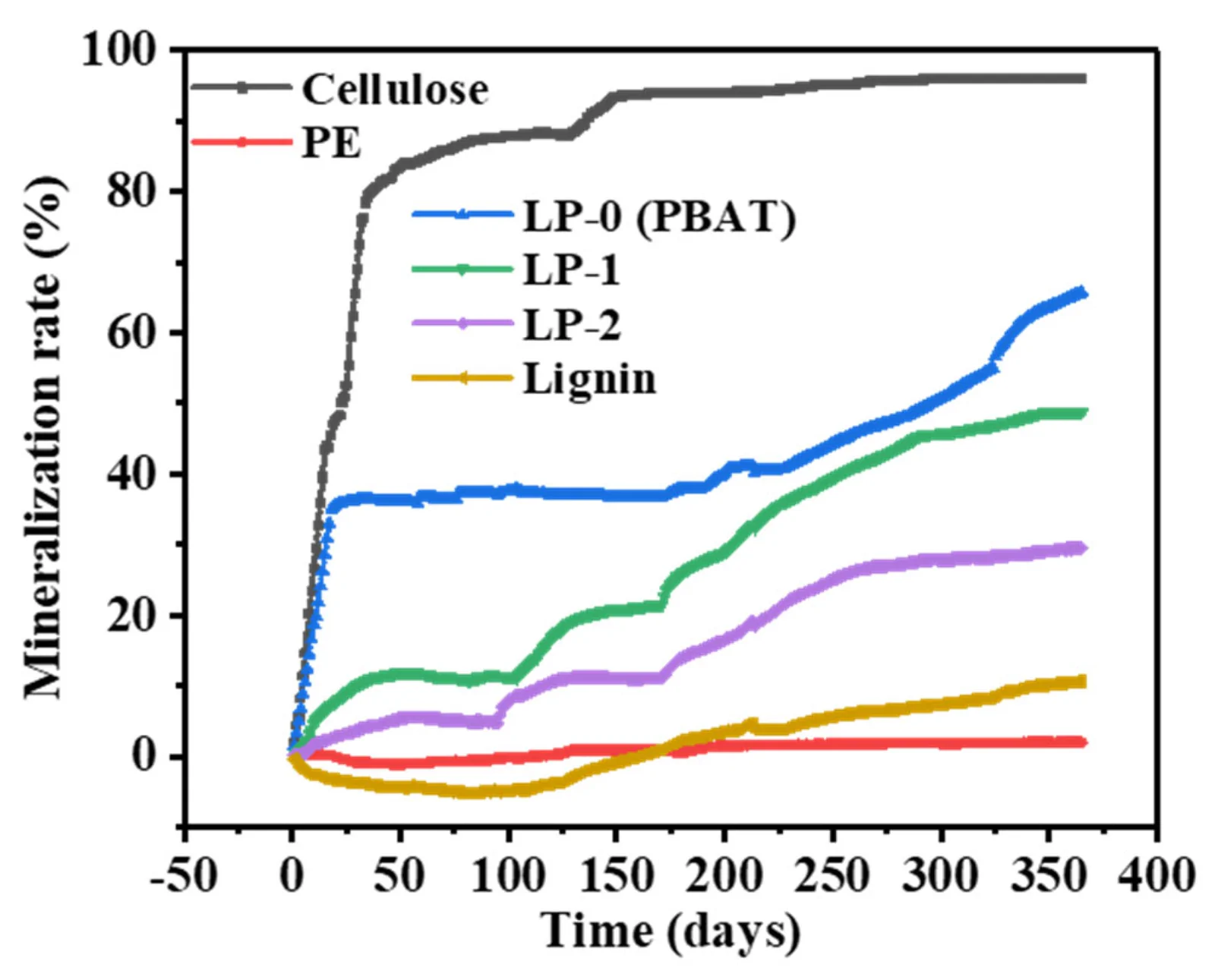
Mineralization rate curves of lignin/PBAT composite powder during composting.

The degradation behavior of lignin/PBAT composites in a composting environment was comprehensively evaluated by two indicators—weight loss rate (film) and mineralization rate (powder)—with the results presented in Figure 4 and Figure 5, which exhibit markedly different trends.

In terms of weight loss, pure PBAT shows less than 1% loss within the first 20 days, whereas LP-1 and LP-2 reach 5.7% and 6.7%, respectively, indicating that the incorporation of lignin significantly accelerates the early-stage hydrolytic weight loss, which may be attributed to the introduction of interfacial regions that enhanced water permeation pathways. By day 40, pure PBAT had disintegrated into extremely fine fragments that were difficult to collect from the compost medium; since the recovered residual mass was effectively zero, the calculated weight loss approached 100% according to Equation (1), reflecting complete physical fragmentation rather than mass loss through mineralization. In contrast, the lignin-containing composite films, owing to their improved tear resistance (consistent with the mechanical property results), maintain larger fragments that can be readily collected and weighed; the weight losses of LP-1 and LP-2 reached 18.3% and 28.6%, respectively, with the rate increasing with lignin content. It should be noted that these values represent the mass loss of the recoverable fragments and may underestimate the true total mass loss since any fine fragments that passed through the sieve were not included. SEM observations of the film surfaces before and after degradation (Figure 4) revealed that all samples became rougher after composting. Specifically, the pure PBAT film displays pitting cavities, which are often associated with microbial hydrolytic attack, whereas the lignin-containing films show a more crack-like surface morphology, which may suggest the occurrence of non-enzymatic hydrolytic degradation pathways. Collectively, these observations suggest that lignin primarily promotes non-enzymatic hydrolysis in PBAT during the early stage, while preserving mechanical integrity against fragmentation at later stages, thereby delaying disintegration into unrecoverable fines.

In stark contrast to the weight-loss trends, the 1-year mineralization experiment reveals a significant inhibitory effect of lignin on ultimate biodegradation. The mineralization rate of cellulose in the compost system reaches 90% at 136 days, confirming that the system maintains high microbial activity throughout the period and ensuring the reliability of the experimental data. Pure PBAT exhibits the fastest mineralization, reaching 65.71% after one year without yet reaching a plateau, while LP-1 and LP-2 achieved only 48.74% and 29.53%, respectively, with the reduction becoming more pronounced as lignin content increases. Notably, the lignin sample shows a negative mineralization rate in the early degradation stage, which may be attributable to the release of phenolic compounds that are known to possess antibacterial activity [37], temporarily suppressing microbial metabolic activity. However, since quantitative structural characterization (e.g., hydroxyl groups and S/G ratio) was not performed in the present study, a direct structure–property relationship cannot be established from the current data. This inhibition appears to be time-dependent: our mineralization data indicate that the suppression is most pronounced within the first 170 days, after which the mineralization rate gradually recovers (Figure 5). Whether this inhibition persists over longer time scales and how gradual detoxification occurs were not addressed in this study and remain open questions for future investigation.

Combining the weight loss and mineralization results, lignin plays a dual role in the PBAT degradation process, promoting hydrolysis while inhibiting mineralization. This seemingly contradictory phenomenon essentially arises from the fact that weight loss primarily reflects physicochemical processes such as non-enzymatic hydrolysis, whereas mineralization rate measures the ultimate carbon conversion driven by microbial enzymatic metabolism. This finding underscores that evaluating degradation performance solely based on weight loss or apparent disintegration is significantly limited; only by incorporating mineralization rate and other carbon-conversion indicators can the full-lifecycle environmental fate of the material be comprehensively assessed. From an application-design perspective, the incorporation of an appropriate amount of lignin (e.g., 1%) can improve the mechanical integrity and degradation controllability of the films without excessively compromising ultimate biodegradability, simultaneously preventing early fragmentation failure and appropriately extending the service life. Therefore, for different application scenarios (such as short-term agricultural mulch films versus long-term packaging materials), flexible balanced design between degradation rate and material performance can be achieved by adjusting lignin content and pretreatment methods.

## 4. Conclusions

In this study, lignin/PBAT composite films with 1 and 3 wt% lignin were prepared and characterized. At 1 wt% loading, the composite retains tensile strength and exhibits slightly improved tear resistance due to uniform dispersion, whereas 3 wt% leads to reduced strength and anisotropic tear behavior due to aggregation and poor interfacial adhesion, as confirmed by SEM. FTIR shows no chemical interactions. Thermally, lignin acts as a nucleating agent, elevating *T*_c_ and *T*_g_, but reduces overall crystallinity; TGA indicates marginal enhancement in thermal stability. Most importantly, composting tests reveal a dual effect: lignin accelerates early hydrolytic weight loss via interfacial pathways, yet significantly inhibits ultimate mineralization (one-year CO_2_ evolution decreases from 65.7% for pure PBAT to 48.7% and 29.5% for LP-1 and LP-2), which is tentatively attributed to the release of phenolic compounds with potential antibacterial activity [37]. These findings underscore that weight loss alone cannot reflect true biodegradability; mineralization rate is essential. Overall, based on the laboratory-scale data obtained in this study, 1 wt% lignin offers a practical balance of mechanical integrity, thermal performance, and degradation controllability without severely compromising ultimate biodegradability, providing a strategy for tailoring PBAT films for applications such as agricultural mulch films and short-term packaging materials. We acknowledge that the present study did not determine the functional group contents (phenolic OH, aliphatic OH, and carboxyl), nor did we compare its properties with those of other lignins. These molecular-level features may influence the antibacterial activity and mineralization behavior, and their investigation remains an important direction for future work.

## Figures and Tables

**Table 1:** The composition of lignin/PBAT composites.

Sample	Lignin [g]	PBAT [g]	Thickness [µm]
LP-0	0	1000	23.9
LP-1	10	1000	22.6
LP-2	30	1000	22.2

**Table 2:** Mechanical properties of lignin/PBAT composites.

Sample	Tensile Strength [MPa]		Tensile Strain [%]		Right-Angled Tearing Load [N]	
	Vertical	Horizontal	Vertical	Horizontal	Vertical	Horizontal
LP-0	28.4 ± 1.2	19.8 ± 0.9	409.2 ± 18.7	656.8 ± 12.2	2.3 ± 0.16	3.7 ± 0.14
LP-1	28.4 ± 2.5	19.8 ± 0.6	485.7 ± 10.2	511.3 ± 24.6	2.6 ± 0.11	4.1 ± 0.18
LP-2	23.9 ± 2.6	16.3 ± 1.2	403.1 ± 5.4	575.5 ± 13.9	2.5 ± 0.15	2.9 ± 0.22

**Table 3:** DSC and TGA results of lignin/PBAT composites.

Samples	DSC					TG	
	*T*_c_ [°C]	Δ*H*_c_ [J·g^−1^]	*T*_g_ [°C]	*T*_m_ [°C]	Δ*H*_m_ [J·g^−1^]	*T*_d_ (95%) [°C]	*T*_max_ [°C]
LP-0	48.4	11.6	−28.5	119.8	17.6	374.9	405.9
LP-1	92.6	10.9	46.4	124.0	11.6	375.3	407.2
LP-2	91.8	10.6	49.1	127.8	13.9	374.3	407.0

Note: The crystallization temperatures (*T*_c_) and the crystallization enthalpy (Δ*H*_c_) were determined from the cooling ramp. The glass transition temperature (*T*_g_), melting temperatures (*T*_m_), and corresponding melting enthalpy (Δ*H*_m_) were obtained from the second heating cycle. The thermal decomposition temperature (*T*_d_ (95%)) is the temperature at which a weight loss of 5% occurs. The temperature at the maximum degradation rate (*T*_max_) was determined by the first derivative thermogravimetric (DTG) curves.

## Data Availability

The original contributions presented in this study are included in the article. Further inquiries can be directed to the corresponding authors.
